# Multiplexed Integrating Plasmids for Engineering of the Erythromycin Gene Cluster for Expression in Streptomyces spp. and Combinatorial Biosynthesis

**DOI:** 10.1128/AEM.02403-15

**Published:** 2015-11-13

**Authors:** Bahgat Fayed, David A. Ashford, Amal M. Hashem, Magdy A. Amin, Omaima N. El Gazayerly, Matthew A. Gregory, Margaret C. M. Smith

**Affiliations:** a Department of Biology, University of York, York, United Kingdom; b Bioscience Technology Facility, Department of Biology, University of York, York, United Kingdom; c Chemistry of Natural and Microbial Products Department, National Research Centre, Cairo, Egypt; d Department of Microbiology and Immunology, Faculty of Pharmacy, Cairo University, Cairo, Egypt; e Department of Pharmaceutics and Industrial Pharmacy, Faculty of Pharmacy, Cairo University, Cairo, Egypt; f Isomerase Therapeutics, Science Village, Chesterford Research Park, Cambridge, United Kingdom

## Abstract

Bacteria in the genus Streptomyces and its close relatives are prolific producers of secondary metabolites with antibiotic activity. Genome sequencing of these bacteria has revealed a rich source of potentially new antibiotic pathways, whose products have never been observed. Moreover, these new pathways can provide novel genes that could be used in combinatorial biosynthesis approaches to generate unnatural analogues of existing antibiotics. We explore here the use of multiple orthologous integrating plasmid systems, based on the *int/attP* loci from phages TG1, SV1, and ϕBT1, to express the polyketide synthase (PKS) for erythromycin in a heterologous Streptomyces host. Streptomyces strains containing the three polyketide synthase genes *eryAI*, *eryAII*, and *eryAIII* expressed from three different integrated plasmids produced the aglycone intermediate, 6-deoxyerythronolide B (6-dEB). A further pair of integrating plasmids, both derived from the ϕC31 *int/attP* locus, were constructed carrying a gene cassette for glycosylation of the aglycone intermediates, with or without the tailoring gene, *eryF*, required for the synthesis of erythronolide B (EB). Liquid chromatography-mass spectrometry of the metabolites indicated the production of angolosaminyl-6-dEB and angolosaminyl-EB. The advantages of using multiplexed integrating plasmids for engineering expression and for combinatorial biosynthesis were demonstrated.

## INTRODUCTION

Actinobacteria, such as those in the genus Streptomyces and its close relatives, are rich sources of antibiotics and other bioactive compounds. Indeed, 70% of antibiotics in current use are derived from or inspired by secondary metabolites from these bacteria. In order to meet the growing need for new antibiotics, researchers are turning to new ways of bioprospecting (1, 2). A promising approach is to exploit the multiple “silent” secondary metabolism pathways within actinobacterial genomes, to “wake up” the pathway and identify a new natural product. Genes from these new pathways can also be used in combinatorial biosynthetic approaches with known pathways to generate novel compounds (3). While cloning entire secondary metabolic pathways into heterologous hosts is now generally feasible (4, 5), the tools that facilitate combinatorial biosynthesis and synthetic biology approaches are less well developed.

Phage-encoded integrases have been widely used in integrating vectors for gene cloning in the actinobacteria and especially in Streptomyces (6, 7). The vectors are convenient to use since they are easily maintained and genetically modified in Escherichia coli and can then be transferred by conjugation to Streptomyces, where they integrate into the chromosome at high efficiency. The plasmid size for transfer to Streptomyces appears to be limited more by their stability and maintenance in the E. coli donor strain rather than the ability to establish and integrate in the Streptomyces chromosome. The advantage of using integrating plasmids includes the stability conferred by the directionality of phage-encoded integrases (8). The integration reaction involves a single DNA crossover between the phage attachment site, *attP* and the bacterial attachment site, *attB*, to generate the integrated DNA flanked by the recombinant sites, *attL* and *attR*. As the excision reaction, in which *attL* and *attR* recombine to regenerate *attB* and *attP*, can only occur in the presence of the recombination directionality factor (RDF), plasmid vectors based solely on the *int/attP* locus remain stably integrated (9). Another major advantage of using integrating plasmids is that they can be multiplexed without interference during recombination or conjugation and without any loss of stability, as shown previously by the introduction of ϕC31 and ϕBT1 based plasmids into S. coelicolor and S. lividans (10). Recently, two more phage-encoded *int/attP* loci, from TG1 and SV1, have been described for use in integrating plasmids into Streptomyces genomes (11, 12). These four orthologous integration loci potentially provide a means for efficient combinatorial engineering of antibiotic pathways.

**FIG 1:**
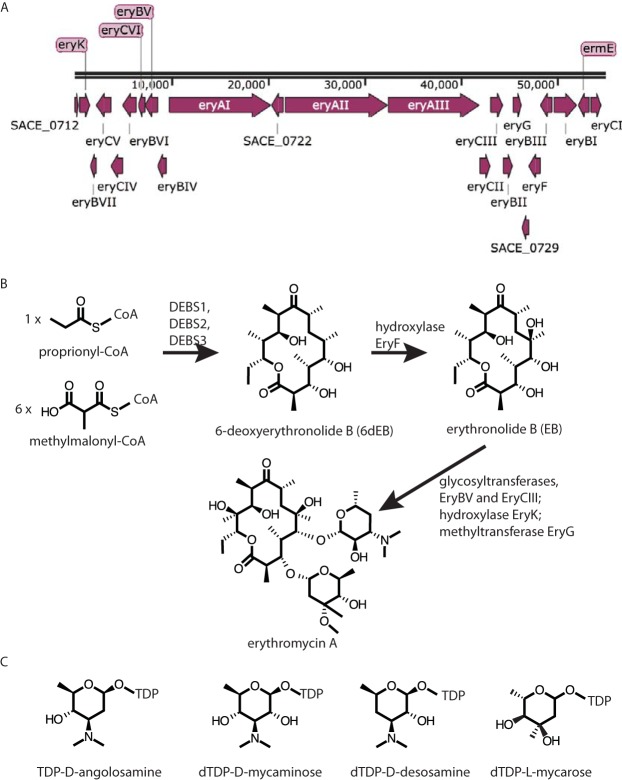
Overview of the biosynthesis of erythromycin. (A) Map of the erythromycin gene cluster from S. erythraea NRRL 2338. (B) Synthesis of erythromycin A showing the intermediates 6-deoxyerythronolide B (6-dEB) and erythronolide B (EB). (C) Four activated sugars relevant to the present study.

To exemplify the use of multiplexed integrating vectors to facilitate genetic manipulation and combinatorial biosynthesis of antibiotic pathways, we chose the erythromycin biosynthesis cluster (Fig. 1A). Erythromycin A is a bacteriostatic macrolide antibiotic produced from Saccharopolyspora erythraea (formerly Streptomyces erythreus) (13). The biosynthesis of erythromycin can be divided into two stages (14). First, the modular polyketide synthase (PKS) complex, 6-deoxyerythronolide B (6-dEB) synthase, catalyzes the sequential condensation of proprionyl-coenzyme A (CoA) and six methylmalonyl-CoA precursors to generate 6-dEB, the first isolatable intermediate in the pathway (Fig. 1). The second stage is the conversion of 6-dEB to erythromycin A, starting with the conversion of 6-dEB to erythronolide B (EB) by EryF hydroxylase (15). Two deoxysugars are then transferred to the aglycone ring to generate the first bioactive intermediate, erythromycin D (15); EryBV glycosyltransferase transfers l-mycarose to yield 3-*O*-mycarosylerythronolide B and then EryCIII, activated by EryCII, transfers d-deoxydesosamine to the C-5 hydroxyl (14, 16). The genes required for the biosynthesis of the activated sugars, TDP-deoxymycarose and TDP-deoxydesosamine, are all encoded within the erythromycin gene cluster (16). The final two tailoring steps, hydroxylation of C-12 by EryK hydroxylase (17), and methylation the 3′-OH of mycarose by EryG methyltransferase lead to the final product erythromycin A (18) (Fig. 1).

The biosynthetic pathway of erythromycin A offers multiple opportunities for combinatorial biosynthesis and the production of unnatural analogues. Genetic manipulation of such a large cluster comprising 55 kbp and 22 genes represents a daunting challenge (Fig. 1) (19). Engineering the erythromycin gene cluster in the native strain presents further difficulties due to the fastidious nature of S. erythraea and the low frequency of transformation by large plasmids (20). Despite these, previous researchers have been immensely successful at rational alteration of the pathway to produce new analogues of the intermediate 6-dEB (21–23). Several of these studies have relied on expression in a heterologous host, S. coelicolor, S. lividans, or E. coli (24). Using a freely replicating SCP2*-derived plasmid encoding all three of the PKS enzymes (DEBS1, DEBS2, and DEBS3, encoded by *eryAI*, *eryAII*, and *eryAIII*), Kao et al. was able to express 6-dEB and 8,8a-deoxyoleandolide in S. coelicolor (21). Further work by McDaniel et al., using the same genetic construct as used by Kao et al., demonstrated that the aglycone ring could be modified by substitution of enzyme domains from the homologous rapamycin pathway to produce 61 6-dEB analogues (25). At the same time, Xue et al. placed the three *eryA* genes on different plasmids using SCP2* for *eryAI* and *eryAII* and the ϕC31 *int/attP* site for *eryAIII* (26). Since two of the plasmids are derived using the same replicon, SCP2*, selection had to be constantly applied to ensure against plasmid loss (26). Despite this and other potential problems that were predicted to arise through the use of vectors derived from the same incompatibility group Xue et al. demonstrated the principle of in *trans* expression of the PKS genes in the heterologous host. Moreover, placing the three *eryA* genes on different plasmids greatly facilitated combinatorial engineering of the *eryAI*, *eryAII*, and *eryAIII* genes.

Here, we adapt and improve the strategy taken by Xue et al., and we show that expression of the *eryAI*, *eryAII*, and *eryAIII* genes can be achieved from three orthologous integrating vectors in S. coelicolor. Using a fourth plasmid, we demonstrate how the tailoring steps downstream of the synthesis of 6d-EB and EB might be easily modified.

## MATERIALS AND METHODS

### Bacterial strains and culture conditions.

E. coli strain DH5α, E. coli strain ET12567(pUZ8002), and E. coli strain BW25113(pIJ790) were grown as described elsewhere (27, 28). E. coli strain DH5α was used for plasmid propagation and subcloning while E. coli strain BW25113(pIJ790) was used for recombineering using the REDIRECT method (27). The DNA methylation deficient strain of E. coli, ET12567(pUZ8002) was used as the donor host for plasmid conjugation to Streptomyces species as described previously (29).

Streptomyces strains were maintained on Soya Mannitol (SM) agar at 30°C (30). Streptomyces strains used as heterologous expression hosts were Streptomyces coelicolor J1929, a Δ*pglY* derivative of the wild-type strain M145 (31), Streptomyces coelicolor M512 (Δ*redD ΔactII-orf4* SCP1^−^ SCP2^−^) (32), Streptomyces coelicolor M1152 [Δ*act* Δ*red* Δ*cpk* Δ*cda rpoB*(*C1298T*)], Streptomyces coelicolor M1154 (Δ*act* Δ*red* Δ*cpk* Δ*cda rpoB*(*C1298T*)], *rpsL*(*A262G*) (33), and Streptomyces lividans TK24 (*str-6* SLP2^−^ SLP3^−^) (30). Micrococcus luteus (Fleming strain) was grown in tryptic soy broth (TSB) at 30°C and was used as a challenge organism for the detection of antibiotic activity.

### DNA manipulations.

Chemically competent E. coli cells were prepared, stored, and used in the transformation procedure as described previously (28). Plasmid DNA extraction from E. coli was performed using QIAprep spin miniprep kit according to the protocol supplied by the manufacturer (Qiagen). Restriction enzymes used during the study were obtained from New England BioLabs (NEB), and the digestion procedure was carried out according to the manufacturer's instructions. Phusion high-fidelity DNA polymerase (NEB) was used for PCR amplification unless otherwise stated. The primers used in the present study are listed in Table 1. Overlap extension PCR was used to attach two or more DNA fragments when required, as described previously (34). An In-Fusion HD cloning kit (Clontech) was used generally for cloning DNA fragments according to the protocol supplied by the manufacturer.

**TABLE 1:**
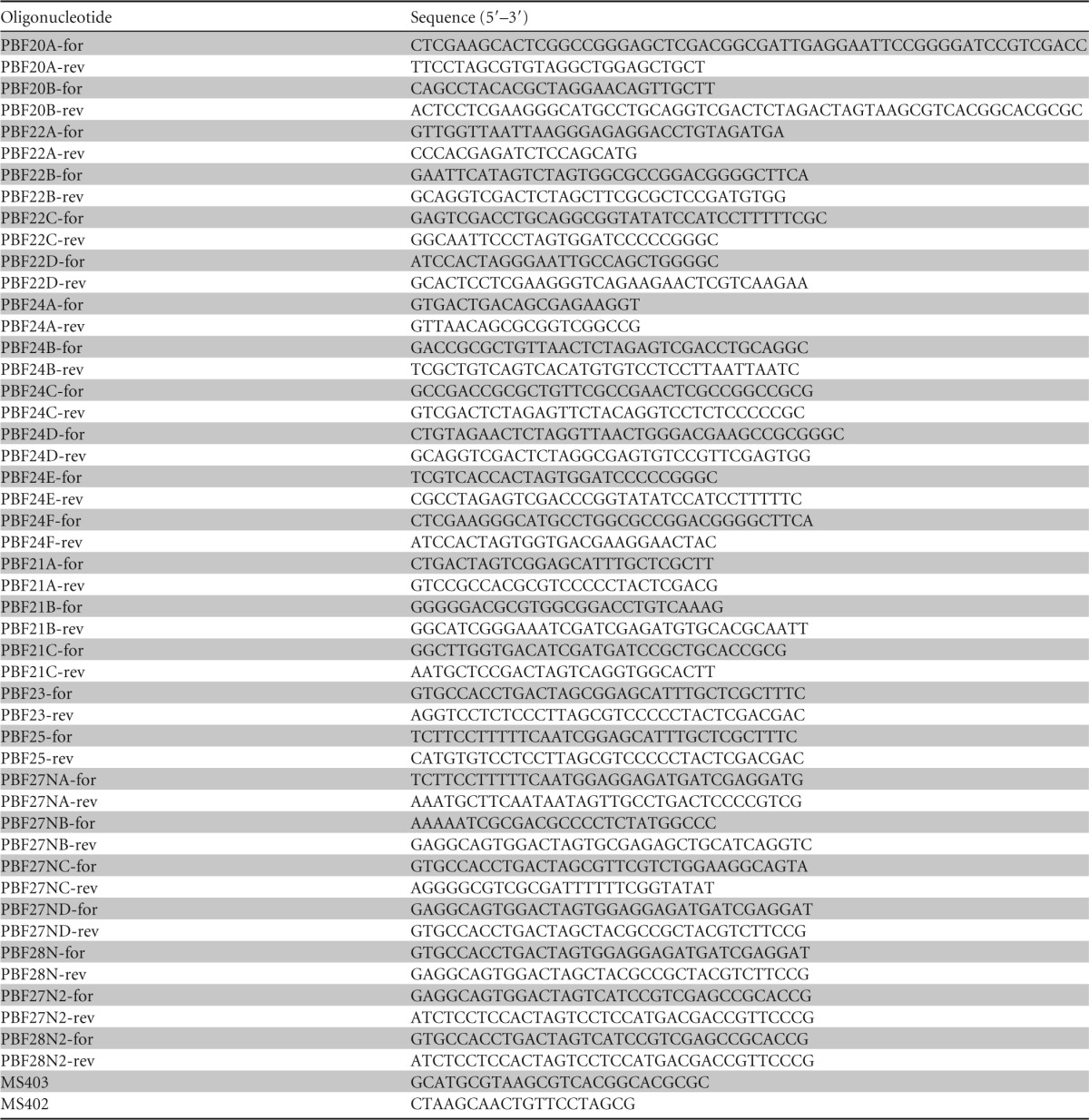
Oligonucleotides used in this study

### Plasmid constructions. (i) Construction of pRF10.

The *int/attP* region of the phage TG1 was amplified by PCR using TG1 genomic DNA as a template and the primer MS403. The amplified product fragment was then digested using SpeI and SphI and ligated using NEB quick ligation kit to the SphI/SpeI 1,994-bp fragment from pMS82 to generate pRF10. The ligation mixture was introduced into E. coli by transformation and used in a conjugation assay to verify efficient integration of pRF10 into S. coelicolor, S. lividans, S. venezuelae, S. avermitilis, and S. albus.

### (ii) Construction of the expression plasmid pBF20.

**FIG 2:**
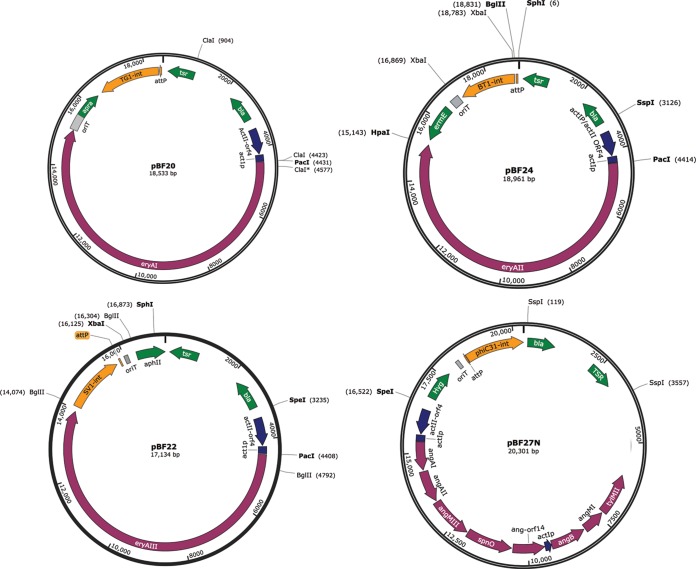
Plasmid constructs for the expression of 6dEB and the angolomycin glycosylation gene cassette.

pBF20 (Fig. 2) is an integrating plasmid derived from phage TG1 *int/attP* and contains the *eryAI* gene under the control of the *actI*p promoter and the apramycin-resistant gene (*aac(3)IV*) for selection. pBF20 is a derivative of pIB023 (see Fig. S1 in the supplemental material), a plasmid containing the *eryAI*, *eryAII*, and *eryAIII* genome region from the native erythromycin cluster. pIB023 was created by insertion of pCJR65 into the S. erythraea genome, followed by rescue the large EcoRI fragment containing all of the *eryA* genes (35). Upstream of *eryAI* in pIB023 is the *actI*p promoter and the *actII-orf4* gene encoding the activator of *actI*p (35). The recombineering procedure of Gust et al. (27) was used to replace all of the DNA from the 3′ end of *eryAI* to the 3′ end of *eryAIII* in pIB023 with an overlap extension PCR product encoding *aac(3)IV-oriT*-TG1 *int/attP* (34) generated from two PCR products. The *aac(3)IV-oriT* disruption cassette was amplified by PCR using pIJ773 as the template and primers PBF20A-for and PBF20A-rev. The TG1 *int/attP* locus was amplified using plasmid pRF10 with the primers PBF20B-for and PBF20B-rev. The primers PBF20A-for and PBF20B-rev were then used to create a single amplified product of 3,529 bp.

Primers PBF20A-for and PBF20B-rev contained 39 nucleotides (nt) of sequence that were identical to the endpoints of the DNA to be retained in pIB023 and were necessary to mediate recombineering between the *aac(3)IV-oriT*-TG1 *int/attP* PCR overlap extension product and pIB023 to remove the *eryAII* and *eryAIII* genes. Recombineering was performed by electroporation of the 3,529-bp PCR fragment into E. coli strain BW25113(pIJ790, pIB023) after induction of the *gam*, *red*α, and *red*β genes by arabinose (27). Selection for apramycin resistance yielded the desired plasmid, pBF20 (Fig. 2), which was confirmed by restriction analysis and nucleotide sequencing.

### (iii) Construction of the expression plasmid pBF22.

pBF22 is an SV1 *int/attP*-based integrating plasmid (11) containing the *eryAIII* gene under the control of *actI*p promoter and encodes the kanamycin-resistant gene (*aphII*) for selection (Fig. 2). To generate pBF22, pIB023 was digested with BglII and PacI, and the 13,582-bp DNA fragment was purified. This DNA fragment contains a 5′ truncated *eryAIII* gene downstream of the *actI* promoter, the thiostrepton resistance gene (*tsr*), the ampicillin resistance gene (*bla*), and the E. coli colE1 plasmid origin of replication. The 390-bp region encoding the 5′ end of the *eryAIII* gene was amplified by PCR using pIB023 as a template and the primers PBF22A-for and PBF22A-rev and digested with BglII and PacI. The 13,582-bp fragment and the 390-bp fragments were ligated together to form plasmid pBF22a. The SV1 *int/attP* locus was amplified by PCR using pBF3 and the primers PBF22B-for and PBF22B-rev and inserted by In-Fusion cloning into pBF22a cut with XbaI to give plasmid pBF22b. The *aphII* and *oriT* fragments were amplified separately by using the primer pairs PBF22C-for/PBF22C-rev and PBF22D-for/PBF22D-rev, respectively, and the templates pNRT4 (10) and pIJ778 (27), respectively. Overlap extension PCR was then used with the primers PBF22D-for and PBF22C-rev to fuse the *aphII* and *oriT* PCR fragments to generate a 1,259-bp fragment, which was inserted by In-Fusion cloning into pBF22b linearized with SphI. The final plasmid, pBF22, was confirmed by restriction analysis and nucleotide sequencing.

### (iv) Construction of the expression plasmid pBF24.

pBF24 is ϕBT1 *int/attP*-based integrating plasmid (10) harboring the *eryAII* gene downstream of the *actI*p promoter and containing *ermE* for selection (Fig. 2).

pBF24 was created in multiple steps. A 4,079-bp fragment encoding the 5′ part of *eryAII* was amplified using pIB023 as a template and the primers PBF24A-for and PBF24A-rev introducing a HpaI restriction site at the 3′ end (PBF24A-rev). A 4,475-bp fragment encoding the plasmid vector sequence (containing *bla*, *tsr*, the *actI*p promoter, *actII-orf4*, and the *colE1* origin) was also amplified from pIB023 using the primers PBF24B-for and PBF24B-rev. The 4,079- and 4,475-bp fragments were then ligated together by infusion to generate pBF24A. The remaining 3′ part of the *eryAII* gene was amplified by using the primers PBF24C-for and PBF24C-rev and ligated with pBF24A cut with HpaI to form plasmid pBF24B. pBF24C was constructed by amplification of the *ermE* gene from S. erythraea BIOT-4480 using the primers PBF24D-for and PBF24D-rev and inserting it by In-Fusion cloning into pBF24B cut with XbaI. The *oriT* (amplified using the template pIJ773 and the primers PBF24E-for and PBF24E-rev) and the ϕBT1 *int/attP* (amplified using pMS82 as the template and primers PBF24F-for and PBF24F-rev) loci were joined to generate a 2,312-bp fragment by overlap extension PCR, which was inserted by In-Fusion cloning into pBF24C cut with SbfI. The final plasmid, pBF24, was confirmed by restriction analysis and nucleotide sequencing.

### (v) Construction of the expression plasmids pBF21, pBF23, and pBF25.

Alternative versions of pBF20, pBF22, and pBF24 (Fig. 2), called, respectively, pBF21, pBF23, and pBF25 were made in which the *actII-orf4*/*actI*p promoter was replaced with the native promoter *eryAI*p (see Fig. S2 in the supplemental material).

To construct pBF21, pBF20 was digested with ClaI to yield three DNA fragments of 14,860, 3,521, and 156 bp. An overlap extension PCR product was generated from three PCR products: a 2,381-bp fragment containing the 5′ end of the *tsr* gene, the ColE1 origin, and the *bla* gene; a 240-bp fragment *eryAI*p (amplified from the S. erythraea BIOT-0666 genomic DNA using the primers PBF21A-for and PBF21A-rev); and a 154-bp fragment containing the 5′ end of the *eryAI* gene that was lost after cutting with ClaI. The resulting fragment (2,715 bp, obtained using the primers PBF21C-for and PBF21B-rev) was inserted by In-Fusion cloning into the purified 14,860-bp ClaI fragment to generate pBF21.

For the pBF23, and pBF25 constructions, *eryAI*p was amplified from the S. erythraea BIOT-0666 genomic DNA by primers PBF23-for/PBF23-rev and PBF25-for/PBF25-rev, respectively. The *eryAI*p fragments were then inserted into pBF22 and pBF24, cut with SpeI/PacI and PacI/SspI, respectively. The new plasmids were verified by restriction analysis and sequencing.

### (vi) Construction of the expression plasmid pBF27N.

pBF27N is an integrating plasmid encoding the ϕC31 *int/attP*, derived from a plasmid containing the full angolosamine biosynthesis gene cassette under the control of the *actI*p promoter (36), a hygromycin-resistant gene (*hyg*), and *oriT* (Fig. 2).

The construction was done as follows: pIB023 was cut with PacI and XbaI, the 4428-bp plasmid backbone fragment (*bla*, *tsr*, and the *actI* promoter) was purified, and the ends were then filled in with DNA polymerase I, large (Klenow) fragment to generate blunt ends for ligation. This blunt ended fragment was then self-ligated using Quick Ligase Enzyme (NEB) to produce pBF27A.

The *hyg* gene encoding hygromycin B phosphotransferase was amplified from plasmid pSMT3-M using the primers PBF27NA-for and PBF27NA-rev and inserted by In-Fusion cloning into pBF27A cut with SspI to form plasmid pBF27B. ϕC31 *int/attP* locus and *oriT* were fused by overlap extension PCR; two amplified fragments were prepared using pSET152 as a template and primers PBF27NB-for/PBF27NB-rev and pIB773 as a template and the primers PBF27NC-for/PBF27NC-rev, and the final fused product was obtained using the primers PBF27NC-for/PBF27NB-rev. The fragment containing ϕC31 *int/attP* and *oriT* (2,410 bp) was inserted into pBF27B cut with SpeI by In-Fusion cloning to produce plasmid pBF27C. Finally, the fragment encoding *hyg* ϕC31 *int/attP* and *oriT* was amplified by PCR using the primers PBF27ND-for and PBF27ND-rev and the template pBF27C and inserted using In-Fusion cloning into the SpeI-cut plasmid constructed by Schell et al. encoding the angolosamine biosynthesis cassette (36), thus generating plasmid pBF27N (Fig. 2).

pBF27N2 (see Fig. S2 in the supplemental material) encodes the *eryF* gene in addition to the angolosamine biosynthesis cassette. *eryF* gene was amplified from S. erythraea BIOT-0666 genomic DNA using the primer pair PBF27N2-for/PBF27N2-rev and inserted into pBF27N and pBF28N cut with SpeI by In-Fusion cloning to form plasmid pBF27N2.

### Production and analysis of 6-dEB.

Five Streptomyces strains were used for 6-dEB production: S. coelicolor J1929, S. coelicolor M512, S. lividans TK24, S. coelicolor M1152, and S. coelicolor M1154. Each strain received either pBF20, pBF22, and pBF24 expressing the *eryA* genes from the *actII-orf4/actI*p activator/promoter or pBF21, pBF23, and pBF25 in which *actII-orf4/actI*p was exchanged for the native *eryAI*p promoter. For each strain, the three plasmids were introduced sequentially by conjugation from E. coli. After introduction of the *eryA* genes into the host strains, 10^6^ spores from three independent lines for each strain were cultured with antibiotics in 25 ml of EVL medium (corn steep solids, 15 g/liter; sucrose, 30 g/liter; ammonium sulfate, 4 g/liter; CaCO_3_ 6 g/liter) as seed culture medium for 3 days at 30°C, and then 1 ml of inoculum was transferred to 25 ml of fermentation medium (soy bean flour, 36 g/liter; corn starch, 36 g/liter; ammonium sulfate, 2.4 g/liter; CaCO_3_, 7.2 g/liter; soybean oil, 5 g/liter) fed with 1.2 ml of 40% glucose and 0.2 ml of propan-1-ol at 30°C for 6 days without added antibiotics. Triplicate samples (0.75 ml) were withdrawn from each flask and added to ethyl acetate–0.1% NH_3_ (0.75 ml), followed by shaking (15 min). After centrifugation at 13,000 rpm for 20 min, the organic solvent fraction was removed, dried, and stored at −20°C.

Dried extract was resuspended in methanol (500 μl), and an aliquot (5 μl) was injected for analysis by high-pressure liquid chromatography (HPLC; Dionex Ultimate 3000) on a reverse-phase halo C_18_ column (2.7 μm; 2.1 by 100 mm; flow rate, 0.2 ml/min; 40°C). Solvents A (0.1% [vol/vol] formic acid in water) and B (0.1% [vol/vol] formic acid in methanol) were mixed to give 40% B initially and held at 40% B upon injection for 0.5 min, and then a linear gradient applied from 40 to 100% B. The solvent was then held at 100% B for 1 min prior to reversion back to 40% B and equilibration for 3.5 min before the next injection. 6-dEB production was identified by its mass spectrum using electrospray ionization mass spectrometry (Bruker maXis UHR-TOF; capillary voltage, 4,000 V; mass range, 50 to 650 *m/z*; positive-ion mode).

For 6-dEB quantification, calibration standards containing a fixed amount of the internal standard, erythronolide B (EB; 100 pmol), and increasing amounts of 6-dEB (0 to 100 pmol) were prepared and analyzed in triplicate in 1-μl injection volumes. Both EB and 6-dEB were provided by Isomerase Therapeutics, purified from fermentations with blocked mutants of S. erythraea. Extracted ion chromatograms (EICs) corresponding to the sodium ion adducts of the molecular ion of 6-dEB (M^+^Na^+^ = 409.2567 *m/z*) and the internal standard EB (M^+^Na^+^ = 425.2516 *m/z*) were plotted with a tolerance of ±0.1 *m/z* using Compass DataAnalysis (Bruker). The peak areas were integrated from the EICs using version 3.0 of the Find algorithm in DataAnalysis with a sensitivity of 50% and absolute intensity threshold of 1,000 counts. These peak area values were entered directly into an Excel spreadsheet to calculate peak area ratios between 6-dEB and EB.

In the sample extractions, EB was added as an internal standard. EB was prepared (10 mM solution) and then diluted to 15 μM in solvent (dilution of 45 μl in 30 ml of solvent). EB-spiked solvent (0.75 containing 11.25 nmol) was added to 0.75 ml of culture, and the total was extracted, dried, and resuspended in 500 μl of methanol. Then, 5 μl was used for analysis. The ratio of peak areas for the internal standard to 6-dEB for each sample was determined. The concentration of 6-dEB in the samples was calculated using the calibration curve and the known amount of internal standard present in the sample (113 pmol of EB).

### Production and analysis of erythromycin analogues.

S. coelicolor M512, S. lividans TK24, and S. coelicolor M1152. each containing pBF20, pBF22, and pBF24. were used as recipients for either pBF27N or pBF27N2. The seed cultures, fermentation medium, extraction method, and the liquid chromatography-electrospray ionization-mass spectrometry (LC-ESI-MS) were performed as described for the 6-dEB extractions with the following modifications. Neither seed nor fermentation media contained antibiotics, and the cultures were fermented for 8 days. The Luna C_18_ column (3 μm, 2 by 150 mm) was used for HPLC analysis, instead of halo C_18_ column (2.7 μm, 2.1 by 100 mm), and roxithromycin (2 μM; Sigma-Aldrich) was added to the organic solvent as an internal standard.

### Bioassay of erythromycin analogues.

The antibacterial activity of the erythromycin analogues was evaluated by the agar diffusion method using Micrococcus luteus as the indicator organism (37). Briefly, TSB-agar medium was initially poured into petri dishes and left to solidify. A second portion of TSB-agar containing 50 μl of a Micrococcus luteus overnight culture (grown 18 h at 30°C in Luria-Bertani medium) was added. Aseptically, holes were punched, and then 50 μl of the sample extract was loaded. After 48 h of incubation at 30°C, the growth inhibition zones of Micrococcus luteus were measured and compared to those of the appropriate control.

## RESULTS

### Introduction of integrating plasmids and validation of engineered Streptomyces strains.

Several Streptomyces strains with different genotypes were used as heterologous expression hosts. S. coelicolor J1929 (Δ*pglY* [31]) was used instead of the wild-type strain M145. J1929 is our laboratory standard because it is sensitive to ϕC31 and ϕBT1. S. coelicolor M512 (Δ*redD* Δ*actII-orf4* SCP1^−^ SCP2^−^) (32), S. coelicolor M1152 [Δ*act* Δ*red* Δ*cpk* Δ*cda rpoB*(*C1298T*)], and S. coelicolor M1154 [Δ*act* Δ*red* Δ*cpk* Δ*cda rpoB*(*C1298T*) *rpsL*(*A262G*)] (33) have all been engineered to be good heterologous expression hosts for secondary metabolite pathways. All three strains have mutations that prevent the expression of known pathways, either in pathway specific activators (e.g., M512) or complete pathway deletions (M1152 and M1154). These mutations are thought to increase the flux of precursors to the introduced, heterologous pathway. In addition, S. coelicolor M1152 and M1154 both contain mutations that pleiotropically upregulate antibiotic expression [*rpoB*(*C1298T*) and *rpsL*(*A262G*)] (38–40). Streptomyces lividans TK24 (*str-6* SLP2^−^ SLP3^−^) has also been shown by others to be a useful expression host (41).

The efficiency of conjugation and site-specific integration of the plasmids encoding the erythromycin polyketide synthases or the sugar biosynthesis genes from the angolamycin pathway was routinely between 4 × 10^3^ and 9.5 × 10^5^ per 10^8^ spores regardless of the host strain (see Table S1 in the supplemental material). The days taken to sporulation by the plasmid containing exconjugants were the same as the parental, plasmid-free controls, and there were no observable differences in colony size or pigmentation.

Genomic DNA from the recipient strains was extracted to assay whether the plasmids had successfully integrated into the host chromosomes. A 1,214-bp DNA fragment from *eryAI* gene was amplified by PCR, thus confirming the integration of pBF20 and pBF21. The integration of pBF22 and pBF23 was confirmed by amplification of a 1,969-bp DNA fragment from the SV1 *int/attP* region. A 1,862-bp DNA fragment was amplified from *eryAII* gene to confirm the integration of pBF24 and pBF25. Finally, a 1,297-bp DNA fragment was amplified from the ϕC31 *int* gene to confirm the integration of pBF27N and pBF27N2.

### Production of the erythromycin intermediate 6-dEB.

**FIG 3:**
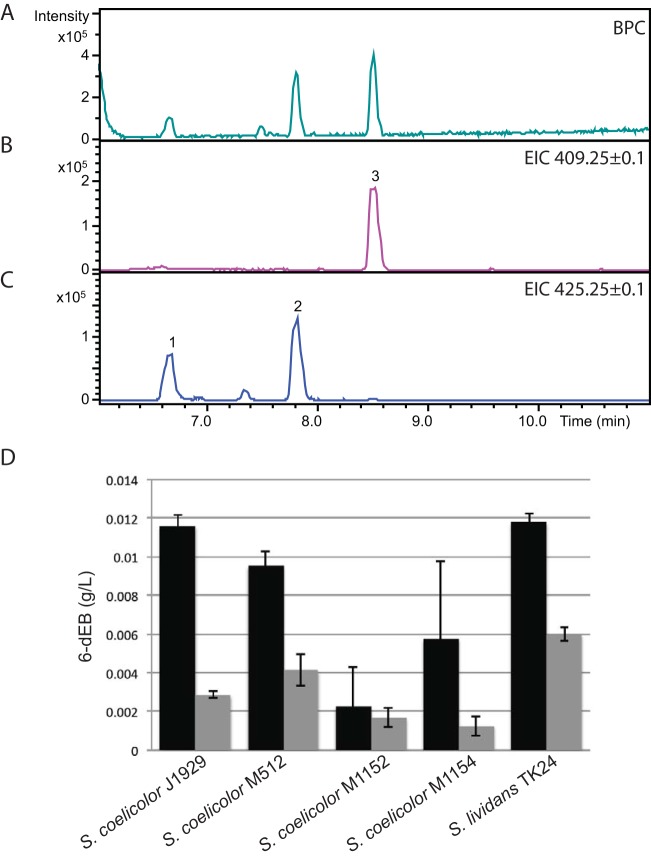
Production of 6-dEB in Streptomyces spp. (A) HPLC base peak chromatogram (BPC) showing the most intense peaks for each mass spectrometry scan in the extract from a Streptomyces lividans TK24::pBF20::pBF22::pBF24 fermentation. (B) Extracted ion chromatogram (EIC) of 6-dEB (*m/z* 409.2; peak 3) from the same extract as in panel A. (C) Extracted ion chromatogram of the internal standard, EB (*m/z* 425.25; peak 2) added to the extract in panel A. The appearance of *m/z* 425.25 at the faster retention time (peak 1) was not observed when the EB standard was analyzed without the extract. (D) Yield of 6-dEB from different Streptomyces strains containing pBF20::pBF22::pBF24 (black bars) or pBF21::pBF23::pBF25 (gray bars). The plasmids pBF20, pBF22, and pBF24 each encode one of the *eryA* genes, with each gene being transcribed from the *actI*p promoter. The plasmids pBF21, pBF23 and pBF25 are derivatives of pBF20, pBF22 and pBF24, respectively, in which the *actI*p promoter has been replaced with the *eryAI*p promoter (gray bars). The results shown are means and standard deviations from between six and nine replicates derived from three biological replicates.

The expected ion (*m/z* = 409.3) indicative of 6-dEB was present in all strains expressing the *eryA* genes from the *act1*p promoter (Fig. 3). Maximum 6-dEB production was from S. lividans TK24::pBF20::pBF22::pBF24 and S. coelicolor J1929::pBF20::pBF22::pBF24 with yields of ∼12 mg/liter. In these fermentations S. coelicolor J1929::pBF20::pBF22::pBF24 and S. lividans TK24::pBF20::pBF22::pBF24 achieved the highest biomasses (0.09 and 0.11 g [dry weight] per 5 ml of culture, respectively) compared to the remaining strains (0.05, 0.055, and 0.06 g [dry weight] per 5 ml of culture for M1154::pBF20::pBF22::pBF24, M1152::pBF20::pBF22::pBF24, and M512::pBF20::pBF22::pBF24, respectively); however, overall, there does not appear to be a simple relationship between the yield and the biomass achieved.

**FIG 4:**
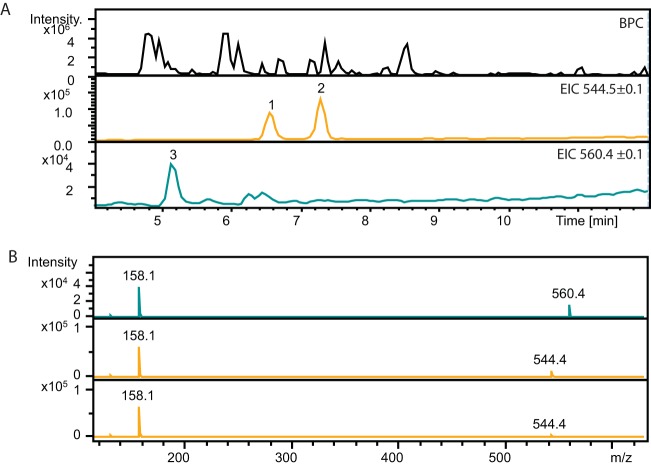
Production of angolosaminyl-6-dEB. (A) HPLC base peak chromatogram (BPC; see legend for Fig. 3) from extracts of S. coelicolor M1152::pBF20::pBF22::pBF24::pBF27N and extracted ion chromatograms (EICs) for the putative angolosaminyl-6-dEB isomers (*m/z* 544.5, peaks 1 and 2; retention times, 6.6 and 7.3 min, respectively) and a putative angolosaminyl-EB (*m/z* 560.4, peak 3; retention time, 5.2 min). (B) MS2 analysis of putative angolosaminyl-EB *m/z* 560.4, (retention time 5.2 min, peak 3; top line) and putative angolosaminyl-6-dEB *m/z* 544.4 isomers (retention times, 6.6 and 7.3 min, peaks 1 and 2, middle and bottom, respectively). All three MS2 analyses show the presence of *m/z* 158.11, which is consistent with all three compounds containing angolosamine.

The production of 6-dEB was also detected from Streptomyces strains containing the plasmids pBF21, pBF23, and pBF25 expressing the *eryA* genes from the native *eryA1* promoter, but the yields were consistently lower than from the *act1*p promoter (Fig. 4); the highest yielding strain (∼6 mg/liter) was S. lividans TK24::pBF21::pBF23::pBF25.

Kao et al. (21) also expressed 6-dEB in S. coelicolor, and these authors also detected a side product, 8,8a-deoxyoleandolide, which is thought to be a product of the DEBS PKS using acetyl-CoA as a starter in place of proprionyl-CoA. We also detected 8,8a-deoxyoleandolide in the fermentation extracts, and the yields were ca. 20% of the yield of 6-dEB. A molecular ion *m/z* = 409.3 indicative of 6-dEB production was not detected in the medium-only control.

### Production of erythromycin analogues in Streptomyces coelicolor.

As our constructs were capable of producing 6-dEB in S. coelicolor, we tested whether we could generate glycosylated derivatives by addition of a fourth plasmid. Schell et al. showed that S. erythraea containing the gene cassette encoding *angAI*, *angAII*, *angMIII*, *spnO*, *ang-ORF14*, *angB*, *angMI*, and *tylMII*, expressed from the *actI*p promoter, could convert exogenously added tylactone to a new compound, 5-*O*-β-d-angolosaminyltylactone (36). We therefore constructed an integrating plasmid, pBF27N, based on the ϕC31 *int/attP* locus and encoding the angolomycin biosynthesis gene cassette (Fig. 3), that could be introduced into the Streptomyces strains producing 6-dEB. In addition, pBF27N was modified to encode EryF, the hydroxylase required for the production of EB from 6-dEB, thus generating pBF27N2. The addition of *eryF* would test whether EB could be produced and provide a second aglycone as a substrate for the heterologous glycosyl transferases encoded in the angolomycin gene cassette, *tylMII/angMIII*.

**FIG 5:**
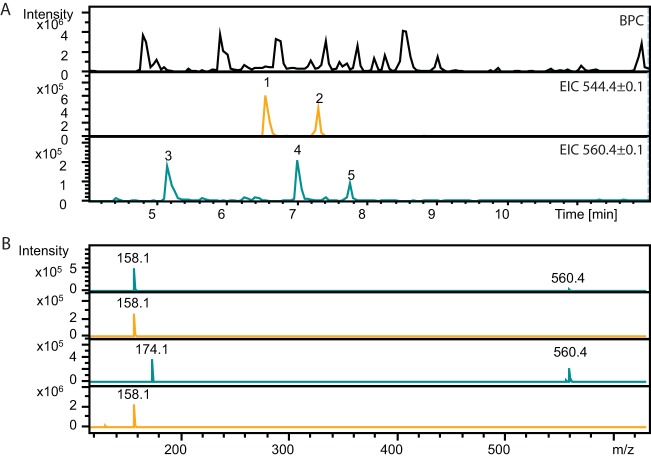
Production of angolosaminyl-6-dEB and angolosaminyl-EB. (A) HPLC base peak chromatogram (BPC; see legend for Fig. 3) in extracts of S. coelicolor M1152::pBF20::pBF22::pBF24::pBF27N2 and extracted ion chromatograms (EICs) for the putative angolosaminyl-6-dEB isomers (*m/z* 544.5, peaks 1 and 2) and *m/z* 560.4, eluting at three retention times (peaks 3, 4, and 5 at 5.2, 7.0, and 7.8 min, respectively). (B) MS2 analysis of the putative angolosaminyl-EB *m/z* 560.4 (peak 3, retention time, 5.2 min) indicated the presence of angolosamine (*m/z* 158.1, top line; see also Fig. 4). MS2 analysis of *m/z* 560.4, retention time 7.0 showed the unexpected presence of *m/z* 174, which suggests that the parent ion could be mycaminosyl-6-dEB (third line down). MS2 analysis of *m/z* 544.4 (peaks 1 and 2, retention times, 6.7 and 7.3 min) indicated that both compounds contain angolosamine (*m/z* 158.1) and are predicted to be angolosaminyl-6-dEB (see also Fig. 4).

LC-ESI-MS was used to detect the expected metabolites in extracts from the fermented cultures (Fig. 4 and 5). S. coelicolor M1152::pBF20::pBF22::pBF24::pBF27N was expected to produce angolosaminyl 6-dEB (*m/z* 544.5), and the corresponding ion was detected but clearly eluting at two retention times (6.6 and 7.3 min; Fig. 4). MS2 analysis of both peaks showed that both contained *m/z* 158.1 consistent with both containing angolosamine. We propose that the two retention times for *m/z* 544.5 correspond to *5-O*-angolosaminyl-6-dEB and *3-O*-angolosaminyl-6-dEB. No ion corresponding to that expected of *bis-O*-angolosaminyl-6-dEB, suggesting that the single glycosylated product cannot act efficiently as a substrate for attachment of a second angolosaminyl residue. Unexpectedly, we observed a very small amount of an ion *m/z* = 560, a retention time of 5.2 min, which could be angolosaminyl-EB (see below), and this was supported by the MS2 data, which indicated that this ion did indeed contain angolosamine. The LC-ESI-MS spectra also indicated the presence of unglycosylated 6-dEB and EB, with peaks at *m/z* 409 and *m/z* 425 (and retention times corresponding to standards for these compounds; Fig. 4). A search for the ion corresponding to angolosaminyl 6-dEB (*m/z* 544.5) in extracts of S. coelicolor M1152::pBF20::pBF22::pBF24 without the angolomycin gene cassette showed no peak higher than the baseline noise, indicating that the glycosylation of 6-dEB was dependent on the presence of pBF27N.

S. coelicolor M1152::pBF20::pBF22::pBF24::pBF27N2 was expected to produce angolosaminyl-EB, with a predicted *m/z* of 560. This ion was observed at three retention times: 5.2 min, 7.01 min, and a very small peak at 7.8 min (Fig. 5). MS2 indicated the presence of *m/z* 158.1 from the peak at 5.2 and *m/z* 174.1 from the peak at 7.01 min, suggesting the presence of angolosaminyl-EB and, possibly, mycaminosyl-6-dEB, respectively (Fig. 5). Mycaminose only differs from angolosamine by the presence of an additional hydroxyl group, explaining the mass differences observed in the MS analysis (Fig. 1 and 5). Intermediates in the production of angolosaminyl-EB were also observed as before, i.e., angolosaminyl-6-dEB isomers were both detected (*m/z* 544.5; 6.6 and 7.3 min, Fig. 5), and both were shown by MS2 to contain angolosamine. A search for *m/z* 560 in extracts of S. coelicolor M1152::pBF20::pBF22::pBF24 without the angolomycin gene cassette did show a peak higher than the baseline noise but still 100-fold lower intensity than those attributed to angolosaminyl-EB and the putative mycaminosyl-6-dEB, indicating that these metabolites were dependent on the presence of pBF27N2.

Without purified angolosaminyl-6-dEB or angolosaminyl-EB standards, the yields of the analogues could not be accurately determined, but we used relative signal intensities and the internal standard, roxithromycin, as a guide to estimate the relative amounts of compounds obtained from the fermentations. The yield of 6-dEB in the M1152::pBF20::pBF22::pBF24::pBF27N cultures (six replicates) was ∼3 mg/liter, and the angolosaminyl-6-dEB isomers amounted to approximately one-tenth to one-fifth of the yield of 6-dEB. The extracts from M1152::pBF20::pBF22::pBF24::pBF27N2 (three replicates) contained ∼8 and 3 mg/liter of 6-dEB and EB, respectively. The yields of angolosaminyl 6-dEB or angolosaminyl EB were estimated to be ∼3-fold less than those observed for 6-dEB and EB. The signal for the putative mycaminosyl-6-dEB was less that one-tenth of that for 6-dEB.

Only traces of *m/z* 544, corresponding to angolosaminyl-6-dEB, were detected in S. coelicolor M512::pBF20::pBF22::pBF24::pBF27N (data not shown). Both M512 and S. lividans containing pBF20::pBF22::pBF24::pBF27N produced 6-dEB, and S. lividans::pBF20::pBF22::pBF24::pBF27N2 produced both 6-dEB and EB (data not shown).

Schell et al. also generated a gene cassette (containing *tylAI*, *tylAII*, *tylMIII*, *tylB1*, *tyl1a*, *tylMI*, and *tylMII*) for the synthesis and transfer of TDP-mycaminose to aglycone rings in biotransformation experiments (36). The plasmid containing this gene cassette was modified with the ϕC31 *int/attP* site and hygromycin marker and introduced into strain expressing 6-dEB, but no glycosylated erythromycin analogues were observed in the fermentation extracts.

### Plasmid stability in S. coelicolor M1152 producing angolosaminyl-6-dEB angolosaminyl-EB.

To test whether strains containing the four integrating plasmids; pBF20, pBF22, pBF24, and pBF27N were stable in S. coelicolor M1152, spores were grown on Soya Mannitol (SM) agar without antibiotics at 30°C. The spores were harvested, and titers were determined on nonselective SM agar and on selective SM agar containing each of the markers present on the four plasmids. Since there was no significant difference in the spore counts between all five plates for each strain, we conclude that the plasmid integrations are stable (see Table S1 in the supplemental material). As a further test, S. coelicolor M1152::pBF20::pBF22::pBF24 was subjected to two rounds of sporulation without selection, and there was no loss of plating efficiency on any of the selective plates.

### Antibacterial activity in extracts containing glycosylated 6-dEB and EB.

**FIG 6:**
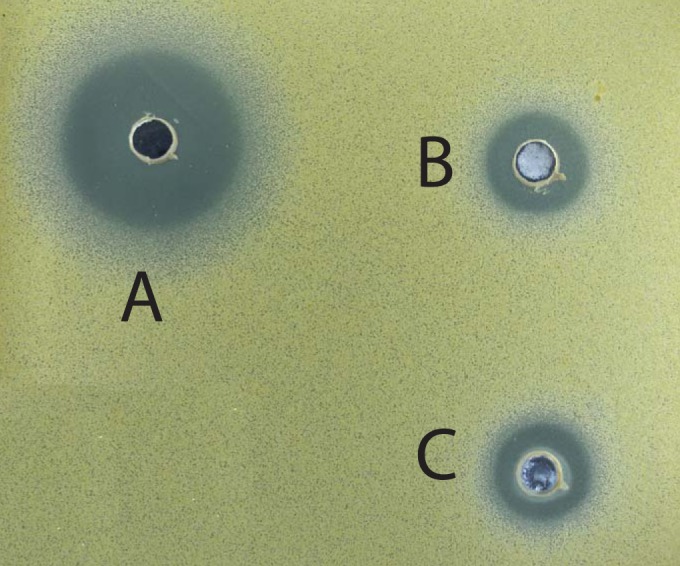
Antibiotic activity in the extracts from S. coelicolor M1152::pBF20::pBF22::pBF24::pBF27N2. Inhibition zones from extracts from S. coelicolor M1152::pBF20::pBF22::pBF24::pBF27N2 (A), M1152::pBF20::pBF22::pBF24::pBF27N (B), and for plasmid-free M1152 (C). This is a representative plate from three independent experiments.

It is known that unglycosylated macrolides from the erythromycin pathway lack antibiotic activity (42). We therefore sought to determine whether the glycosylation of 6-dEB or EB with angolosamine could confer antibiotic activity. Extracts from the fermented cultures of S. coelicolor M1152::pBF20::pBF22::pBF24::pBF27N and M1152::pBF20::pBF22::pBF24::pBF27N2 were dissolved in dimethyl sulfoxide and assayed for the antibacterial activity using a disc diffusion assay. The extract from M1152::pBF20::pBF22::pBF24::pBF27N2 (Fig. 6A) reliably produced a significantly bigger zone of inhibition than those from both M1152::pBF20::pBF22::pBF24::pBF27N (Fig. 6B) and the plasmid free control extract (Fig. 6C). Since both plasmid-containing strains produced mixtures of compounds, including angolosaminyl-EB and angolosaminyl-6-dEB, it was not possible to ascertain the identity of antibiotic itself. However, the antibiotic activity might just be a consequence of higher yield of the glycosylated intermediates produced by M1152: pBF20::pBF22::pBF24::pBF27N2 (see above).

## DISCUSSION

In this study we set out to demonstrate the utility of using multiplexed integrating vectors to engineer antibiotic pathways for expression and combinatorial biosynthesis in a heterologous host. The erythromycin pathway was chosen as an example since it is large, it is well understood, and it still has huge potential for the rational design and expression of analogues. The strategy here was to use four orthogonal integrating vectors based on the Streptomyces phage integrases TG1, BT1, SV1 and ϕC31, each with its cognate *attP* site. These four integrases use different *attB* sites in S. coelicolor or S. lividans, resulting in the stable integration at different genetic loci (8). The genes encoding *eryAI*, *eryAII*, and *eryAIII* genes were inserted into TG1 *int/attP*-, SV1 *int/attP*-, and BT1 *int/attP*-containing plasmids, respectively, while tailoring genes were inserted into the ϕC31 *int/attP*-containing plasmid. This arrangement provides a useful platform for engineering the PKS enzymes and the tailoring enzymes and facilitates combining the engineered constructs once generated.

The advantage of using integration vectors derived using phage integrases is their stability, postintegration, due to the directional properties of the integrases (8, 9). Integrases are able to mediate the integration reaction to generate the prophage but require a second phage-encoded protein, the recombinational directionality factor, or RDF to activate excision. We verified here that the integrated plasmids used in our constructs were stable in the absence of antibiotic selection, and we showed also that fermentations for metabolite production could be performed in the absence of selection (Fig. 3 to 5). The stability of the integrated plasmids improves upon previous work that has utilized replicating plasmids that have required selection for maintenance (21, 26). A number of novel integration systems are becoming available (G. Taylor, P. Fogg, and M. C. M. Smith, unpublished data) that can add to or replace the ϕC31-, TG1-, ϕBT1-, and SV1-derived systems used here. The number of plasmids encoding orthogonal integrases is unlikely to be restrictive in this approach; the upper limit is more likely to be limited by the number of markers available for the selection of exconjugants.

Expression of 6-dEB was robust in the heterologous hosts, with yields varying from ∼1 mg/liter to >10 mg/liter, without any attempt at optimization. The *actI*p*/actII-orf4* promoter/activator once again proved that it is reliable for expression of non-native antibiotic pathways in S. coelicolor and S. lividans. Building on this expression, we generated strains that had the potential to express novel erythromycin analogues. Schell et al. previously constructed gene cassettes for the synthesis of the deoxysugars, d-angolosamine and d-mycaminose (36). S. erythraea lacking the ability to synthesize the aglycone and deoxysugars but expressing the angolosamine cassette is able to transform exogenously added EB to *3-O-*β-D angolosaminyl-EB (36). We used these cassettes to test whether we could generate angolosaminyl or mycaminosyl derivatives of 6-dEB and EB in the heterologous host Streptomyces, into which the genes for aglycone biosynthesis had also been introduced. Two isomers of the expected mass for angolosaminyl-6-dEB were detected, possibly showing that 6-dEB can by glycosylated on either the three- or five-position hydroxyls in the aglycone ring (Fig. 4 and 5). We could also detect a mass consistent with angolosaminyl-EB, and it seems highly likely that this corresponds to the 3-*O*-β-d-angolosaminyl-EB identified by Schell et al. (36).

Some unexpected EB and 6-dEB derivatives were detected. Plasmids pBF27N and pBF27N2 both contain the angolosamine cassette, but only pBF27N2 encodes EryF, the enzyme that introduces the hydroxyl group at C-6 in 6-dEB to generate EB. Despite this, S. coelicolor M1152::pBF20::pBF22::pBF24::pBF27N appears to be synthesizing angolosaminyl-EB in addition to angolosaminyl-6-dEB, the expected product (Fig. 4). The same two products were also observed from S. coelicolor M1152::pBF20::pBF22::pBF24::pBF27N2, which contains *eryF*, but this strain is producing a different side product whose MS spectra are consistent with mycaminosyl-6-dEB (Fig. 5). We cannot at this stage attribute specific host- or plasmid-encoded enzyme activities to these side products.

This angolosamine cassette encodes the *tylMII* glycosyltranferase whose natural activity is to transfer TDP-d-mycaminose to the tylactone ring and is activated by the product of *tylMIII* (36, 43, 44). *tylMII* has been shown previously to have a broad range of substrate specificities, both for the activated sugar and for the aglycone (36, 45). We can therefore add the ability to transfer angolosamine to 6-dEB to the range of TylMII activities. The glycosylation of 6-dEB and EB, however, was not efficient, since only a fraction of 6-dEB and EB were converted. In tylosin biosynthesis, the TylMII glycosyl transferase is normally activated by TylMIII but in pBF27N2, AngMIII, the TylMIII homologue and the activator of AngMII, is used (36, 43). It is not known how compatible this pair of proteins (TylMII/AngMIII) are in mediating the transfer of angolosamine to aglycones. In short, there are viable avenues to pursue to optimize the *in viv*o glycosylation of the aglycone using novel sugar biosynthesis pathways and matching them with different glycosyltransferases.

Extracts producing a mixture of glycosylated analogues, including angolosaminyl-6-dEB, angolosaminyl-EB, and possibly mycaminosyl-6dEB, had antibiotic activity. It is not clear at this stage which of these compounds might have antibiotic activity, and we cannot rule out activation of a cryptic pathway in S. coelicolor (Fig. 6).

Enabling a “plug-and-play” synthetic biology approach to cloning, expression, and modifying antibiotic pathways is a desirable aim, but it is a challenge when the pathways involve many enzymes, including multidomain assembly line proteins such as the PKSs. However, using multiplexed integration vectors to divide and distribute different functional parts of the gene clusters is a useful first step, facilitating combinatorial biosynthesis and the optimization of genetic constructs.

## Supplementary Material

Supplemental material
