# Supplementary material for: Multiplexed Integrating Plasmids for Engineering of the Erythromycin Gene Cluster for Expression in Streptomyces spp. and Combinatorial Biosynthesis
Source: Appl Environ Microbiol. 2015 Nov 13;81(24):8402–13. doi: 10.1128/AEM.02403-15 (PMC4644662; doi:10.1128/AEM.02403-15)
Supplement: Supplemental material [file supp_81_24_8402__index.html]

Supplemental material 

# Multiplexed Integrating Plasmids for Engineering of the Erythromycin Gene Cluster for Expression in Streptomyces spp. and Combinatorial Biosynthesis

## Supplemental material

- Supplemental file 1 -

  Yields of exconjugants per 108 spores (Table S1); spore counts (cfu/ml) for *S. coelicolor* constructs containing 4 plasmids after one round of sporulation without selection (Table S2); plasmid pIB023, carrying the *eryAI*, *eryAII*, and *eryAIII* genes under the control of the *actIp* promoter (Fig. S1); plasmids carrying the *eryAI*, *eryAII*, and *eryAIII* genes under the control of the native *eryAIp* promoter (Fig. S2); plasmid pBF27N2, carrying the angolosamine cassette and the *eryF* gene (Fig. S3).

  PDF, 5.6M
