# Supplementary material for: Multiplexed Integrating Plasmids for Engineering of the Erythromycin Gene Cluster for Expression in Streptomyces spp. and Combinatorial Biosynthesis
Source: Appl Environ Microbiol. 2015 Nov 13;81(24):8402–13. doi: 10.1128/AEM.02403-15 (PMC4644662; doi:10.1128/AEM.02403-15)
Supplement: Supplemental material [file AEM.02403-15_zam999116785so1.pdf]

Fayed et al Supplementary material
Tables S1 and S2.
Figure S1. Map of pIB023
Figure S2. Maps of pBF21, pBF23 and pBF25 encoding *eryAI, eryAII* and *eryAIII* respectively, each with the *eryA1p* promoter upstream.
Figure S3. Map of pBF27N containing the angolosamine cassette and the eryF gene inserted at the SpeI site of pBF27N (shown in Figure 2 in the paper).

Table S1. Yields of exconjugants per 10^8 spores.

	S.coelicolor J1929	S.coelicolor M512	S.coelicolor M1152	S.coelicolor M1154	S.lividans TK24
pBF20	2.7×10^5	5.1×10^4	2.9×10^5	2.3×10^3	1.5×10^5
pBF21	2×10^5	2.4×10^4	1.3×10^5	2.4×10^4	6×10^4
pBF22	2.8×10^4	4.8×10^4	2.8×10^5	2×10^3	5.8×10^4
pBF23	2.1×10^5	2.5×10^4	1.2×10^5	2×10^4	1.8×10^4
pBF24	1.4×10^5	6.3×10^4	4.4×10^4	5×10^3	4.2×10^4
pBF25	2.3×10^5	3.5×10^5	4×10^3	4×10^4	1.9×10^5
pBF30	ND	7.7×10^5	2.4×10^5	ND	3.8×10^5
pBF27N	ND	6×10^5	1.9×10^5	ND	2.8×10^5
pBF27N2	ND	8.5×10^5	2×10^5	ND	1.5×10^5

Table S2. Spore counts (cfu/ml) for *S. coelicolor* constructs containing 4 plasmids after one round of sporulation without selection.

Spore count for *S. coelicolor* M1152: pBF20,:pBF22:pBF24:pBF27N				
SM agar (control)	SM agar (hygromycin)	SM agar (Kanamycin)	SM agar (erythromycin)	SM agar (apramycin)
1.5×10^7	2.1×10^7	1.6×10^7	1.2×10^7	1.2×10^7
Spore count for *S. coelicolor* M1152:pBF20:pBF22:pBF24:pBF27N2				
SM agar (control)	SM agar (hygromycin)	SM agar (Kanamycin)	SM agar (erythromycin)	SM agar (apramycin)
5.9×10^9	4.7×10^9	3.8×10^9	4.7×10^9	4.8×10^9

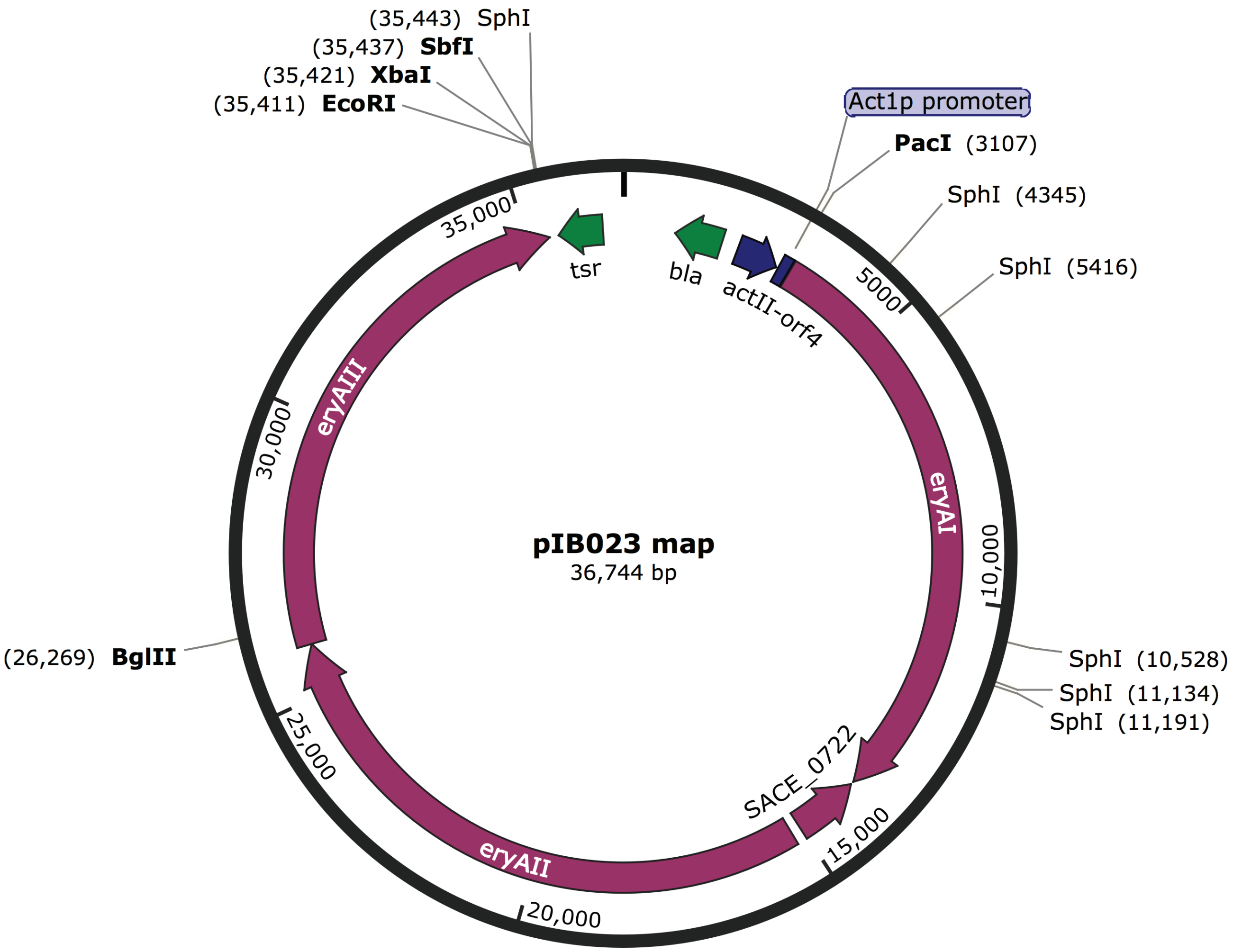

Figure S1. Plasmid pIB023 encoding the *eryAI, eryAII* and *eryAIII* genes under the control of the *actIp* promoter.

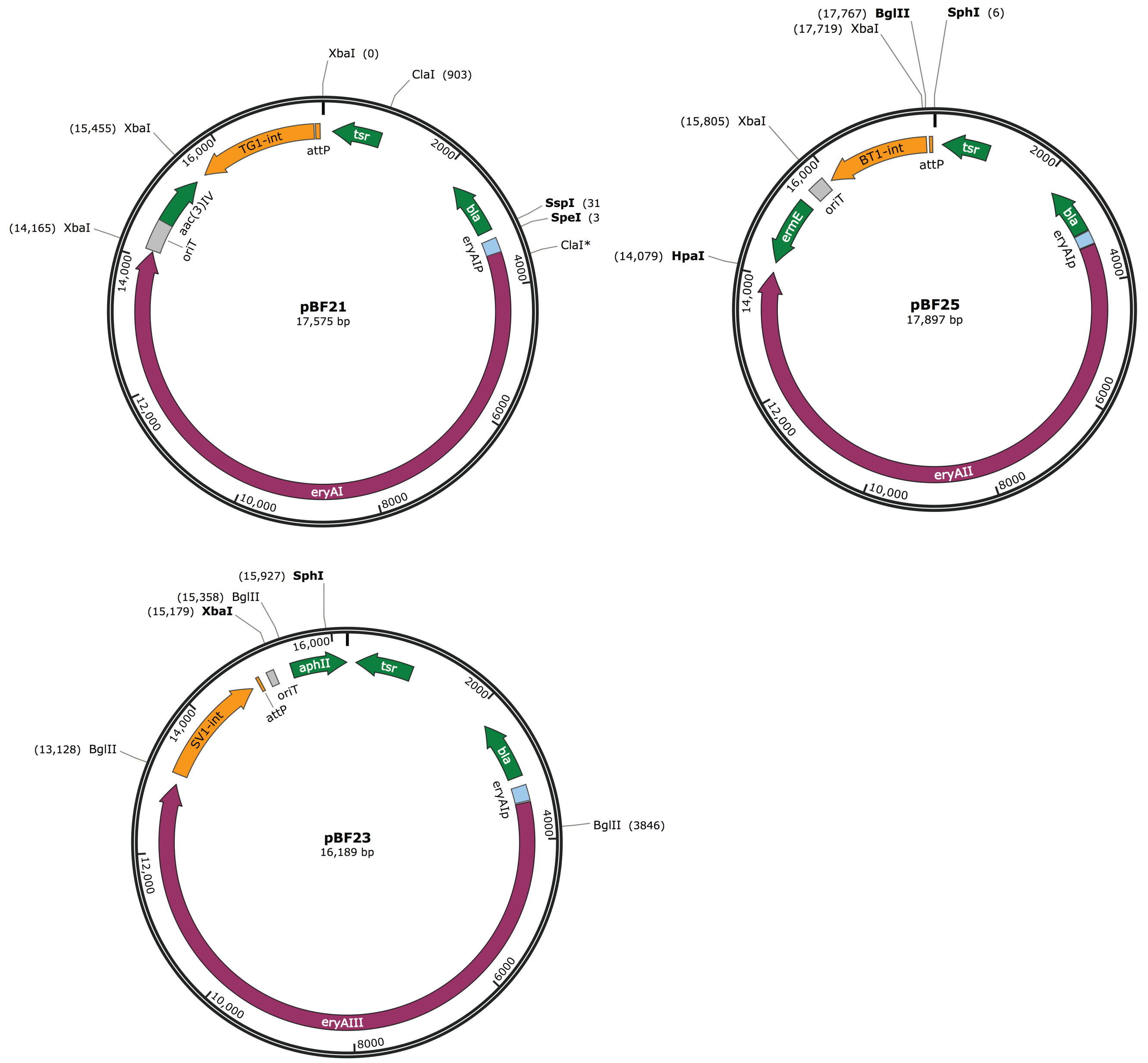

Figure S2. Plasmids encoding the *eryAI, eryAII* and *eryAIII* genes under the control of the native *eryAIp* promoter.

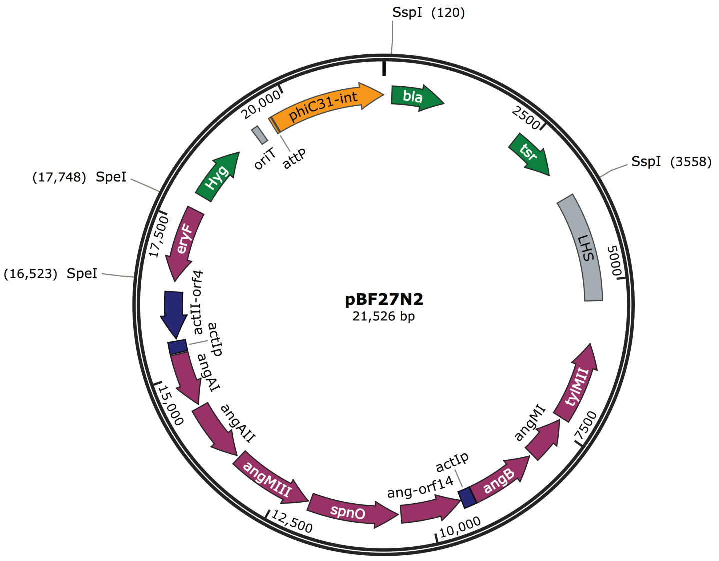

Figure S3. Plasmid pBF27N2 encoding the angolosamine cassette and the *eryF* gene
